# News and Coming Events

**Published:** 1907-06-01

**Authors:** 


### NEWS AND COMING EVENTS.

A three days' bazaar in aid of the funds of the Twerton Infirmary realised a sum of £500.

The Handel Cossham Memorial Hospital at Kingswood, Bristol, will be opened by Mr. Birrell, M.P., on June 1. The hospital has been built and endowed out of a legacy of £110,000 left by the late Mr. Handel Cossham.

Sir Arthur Rucker, M.A., D.Sc., LL.D., F.R.S., Principal of the University of London, will distribute the prizes to the successful students at Guy's Hospital on Thursday, July 4 next. The usual garden party will be given in the grounds.

A festival dinner in aid of the funds of the Mount Vernon Hospital for Consumption and Diseases of the Chest, Hampstead and Northwood, will be held on Monday, June 24, 1907, at the Hôtel Cecil, Strand, W.C. Henry Stedall, Esq., Chairman of the Committee of Management, will preside.

The matron and nursing staff of the Gordon Hospital, Vauxhall Bridge Road, are organising a sale of work in aid of the fund for renovating the wards. The sale will be opened at the hospital on Tuesday, June 11, at three o'clock, by Her Highness the Ranee of Sarawak (Lady Brooke). Princess Victoria and Princess Louise of Schleswig-Holstein have promised to be present.

Under the auspices of Sir William Treloar, Messrs. Bemrose and Sons, Ltd., will publish "Crutches to Help Cripple Children," on June 5. The book will be edited by Sir James D. Linton and Sir Douglas Straight, and has a unique list of contributors in art and letters. The publishers are sparing no efforts to produce it in an attractive manner, and the new colour printing process is being employed to good effect. It will be published at 1s. net, and obtainable through all booksellers.

Commemoration Day of Livingstone College falls on Wednesday, June 5. At 3.30 p.m. on that day a meeting will be held in the college grounds, when short addresses will be given by old students of the college who have been medical missionaries in the Congo, Southern Rhodesia, and India. Sir John Kennaway, Bart., M.P., President of the Church Missionary Society, will take the chair. The college will be open for inspection, and all friends of the institution will be cordially welcomed.

The recently announced death of Dr. Robert Barnes at the advanced age of 90 recalls his singularly varied and distinguished career as the foremost obstetrician of his day. Dr. Barnes was unique in having held appointments on the staffs of three London general hospitals and their medical schools, as well as of five other less prominent institutions. His "Lectures on Obstetric Operations" is the one classic work which no subsequent text-book on midwifery has failed to quote copiously. For many years a prominent contributor to the *Lancet*, his interest in medical literature continued active until very lately; indeed, more than one article from his pen has appeared during the present century in the "Hospital Gazette" of his old school, St. George's. Nor did literature and his own specialty absorb all his energies; throughout life Dr. Barnes was a man of wide culture and many attainments. His death removes a venerable figure from the medical world; a consultant who began work in general practice, his name has always stood for the highest and best ideals in the special line to which he afterwards devoted himself.

Early in the year the committee of the Gateshead Children's Hospital made an appeal to the public for £4,000 to enable them to increase the much needed accommodation at the institution. At present the 22 cots are all occupied, and 21 cases are awaiting admission. The proposed extension will include two large wards of 16 beds each, with two smaller wards, and the neccessary accessories. Already £1,623 12*s*. has been promised.

Last week the staff at the Poole Cornelia Hospital entered into possession of the new premises erected, at a cost of nearly £5,000, in Ringwood Road, Longfleet, almost next door to the Longfleet St. Mary's Church. They had not been in long when the first accident was brought in. The new hospital was formally opened on Thursday, May 23. The old hospital in Market Street, just vacated, is being acquired by the Poole Corporation for municipal offices, etc., at a purchase price of £1,600, and is to be converted into suitable offices at an expenditure of another £1,000. To the great regret of the promoters of the scheme for a new hospital, the public has not subscribed to the building fund as was very reasonably expected they would do, and it is feared that the £1,600 to be received from the Poole Corporation for the old building will have to go to the building fund, and not to the endowment fund, as was very much desired and hoped.

Princess Christian has opened the new sanatorium for consumption at Benenden. The sanatorium, which is situated about six miles from Cranbrook Station, stands about 250 feet above sea-level and faces due south. When completed it will consist of four main sections—a central block of two storeys, accommodating 68 patients, of whom 20 are in single rooms, and the remainder in double-bedded rooms, a number of pavilions, holding ten patients each, an administration block and dining-hall, and a laundry and electric light block. The main building is of steel framing throughout, the space between the girders and stanchions being filled in with "Frazzi," cement rough-cast on the outside and plastered on the inside. This construction has the advantage of being warm in winter and cool in summer, it is fireproof, and it is economical, the total cost for the accommodation of 200 patients at Benenden being £20,000, or £100 per bed, a figure which is much lower than has yet been reached in the case of any other building of the same kind.

Hospital Dinners.—The annual dinner of the Royal Hospital for Incurables was held on May 16, at the Hôtel Metropole, under the presidency of Sir Walter Vaughan Morgan. The hospital dates back to 1854, and its first chairman was Charles Dickens. As a result of the special appeal issued in connection with the dinner a total sum of £3,295 has been contributed towards the funds of the institution.—The Royal Waterloo Hospital's annual dinner took place on the 17th inst. at the Savoy. The Duke of Argyll proposed "Prosperity to the Hospital," and stated that the sum necessary to liquidate the building fund debt and to complete the new building was £35,000. Subscriptions amounting to £1,300 were received.—Baron von Stumm, German Chargé d'Affaires, presided at the 62nd anniversary dinner of the German Hospital, Dalston, which was held at the Hôtel Metropole on May 13. The expenses of the hospital amount to £11,000 a year, and the cost per occupied bed is the lowest in any hospital of the same size in London. Additional accommodation is urgently needed. Subscriptions to the amount of £3,758 were announced at the dinner, including £200 from H.I.M. the German Emperor, and £50 from the Emperor of Austria.

